# Biochemical and Molecular Mechanisms of Folate Transport in Rat Pancreas; Interference with Ethanol Ingestion

**DOI:** 10.1371/journal.pone.0028599

**Published:** 2011-12-06

**Authors:** Nissar Ahmad Wani, Ritambhara Nada, Jyotdeep Kaur

**Affiliations:** 1 Department of Biochemistry, Postgraduate Institute of Medical Education and Research, Chandigarh, Chandigarh, India; 2 Department of Histopathology, Postgraduate Institute of Medical Education and Research Chandigarh, Chandigarh, India; University of South Alabama, united States of America

## Abstract

Folic acid is an essential nutrient that is required for one-carbon biosynthetic processes and for methylation of biomolecules**.** Deficiency of this micronutrient leads to disturbances in normal physiology of cell. Chronic alcoholism is well known to be associated with folate deficiency which is due, in part to folate malabsorption. The present study deals with the mechanistic insights of reduced folate absorption in pancreas during chronic alcoholism. Male Wistar rats were fed 1 g/kg body weight/day ethanol (20% solution) orally for 3 months and the mechanisms of alcohol associated reduced folate uptake was studied in pancreas. The folate transport system in the pancreatic plasma membrane (PPM) was found to be acidic pH dependent one. The transporters proton coupled folate transporter (PCFT) and reduced folate carrier (RFC) are involved in folate uptake across PPM. The folate transporters were found to be associated with lipid raft microdomain of the PPM. Ethanol ingestion decreased the folate transport by reducing the levels of folate transporter molecules in lipid rafts at the PPM. The decreased transport efficiency of the PPM was reflected as reduced folate levels in pancreas. The chronic ethanol ingestion led to decreased pancreatic folate uptake. The decreased levels of PCFT and RFC expression in rat PPM were due to decreased association of these proteins with lipid rafts (LR) at the PPM.

## Introduction

Folate is a member of vitamin B group and is required for the transfer of one carbon unit during nucleic acid synthesis and for metabolism of amino acid [1], [2]. For this reason, cellular deficiency of this essential micronutrient in certain organs leads to disturbances in the normal physiology of the cell that is ultimately manifested in the form of undesirable clinical symptoms. Folate deficiency can prevalent in the underdeveloped countries, and even in the western world. Subtle deficiency is a public health problem that is most notable in its association with neural tube defects in the developing embryo [3]. Due to exogenous requirement of folate in mammals, there exists a well-developed epithelial folate transport system for the intestinal absorption of folate as well as for the regulation of normal folate homeostasis and renal tubular reabsorption [4]–[6]. In contrast to the understanding of the molecular mechanisms of folate uptake by digestive tissues and its excretion by kidneys, not much is currently known about the mechanism and regulation of folate uptake by pancreatic cells [1], [7]. Folate deficiency in pancreas results in disturbances in one-carbon metabolism, which might contribute to the pathogenesis of several pancreatic disorders and might cause cancer [8], [9]. Also folate deficiency results in reduction in amylase secretion and impairs pancreatic secretion [10]–[12]. Thus studying the molecular mechanisms regulating the folate uptake by the pancreatic cells is of physiological, nutritional, and clinical importance.

Folic acid deficiency is often associated with alcoholism worldwide. There is a strong association of alcohol with acute and chronic pancreatitis, affecting 85 per 100 000/year in United States [13]. Long term alcohol abuse is a major risk factor for developing pancreatic disease. Excessive alcohol intake in an experimental animal model exerts a multifaceted impact on the bioavailability and subsequent metabolism of folate and more broadly, on one-carbon metabolism as a whole [14]. Therefore in addition to its direct effects on pancreatic dysfunction, the ethanol might impart its effects through associated folate deficiency and disturbed one carbon metabolism. We have earlier shown the transcription regulation of the folate transporters to be the main molecular mechanism of alcohol associated poor absorption of folate in intestine, colon, and kidney [4], [15]–[20]. Recently, Said et al. [21] have demonstrated molecular mechanisms of folate transport regulation in pancreatic acinar cells during folate deficient conditions and have also shown the reduced mRNA expression of the folate transporters in the pancreatic acinar cells during chronic alcoholism. However, the kinetic behavior of the folate transport systems, molecular mechanisms regulating expression of folate transporters at the protein levels, distinct role of proton coupled folate transporter (PCFT) and reduced folate carrier (RFC) and distribution of folate transporters in lipid rafts in the PPM, under alcoholic conditions have not been evaluated.

We studied the expression of the folate transporters viz. PCFT and RFC, their distribution in lipid rafts across the pancreatic plasma membrane under alcoholic conditions. This study bears significance in view of the fact that these transporters are important determinants for the chemotherapeutic potential of various antifolates. The results of the study indicated the existence of a specialized, acidic pH-dependent folate uptake in PPM, with the involvement of both RFC and PCFT. Ethanol feeding resulted in upregulated expression of the folate transporters (RFC and PCFT) in the pancreas. However there were reduced levels of transporters at the PPM in association with lipid rafts, which is responsible for folate malabsorption across pancreatic plasma membrane in chronic alcohol fed rats. The reduced folate levels in the pancreas of ethanol fed rats was associated with hypomethylation in CpG island of RFC but not of PCFT gene.

## Results

### Transport of 5-methyltetrahydrofolate across pancreatic plasma membrane

**Figure 1:**
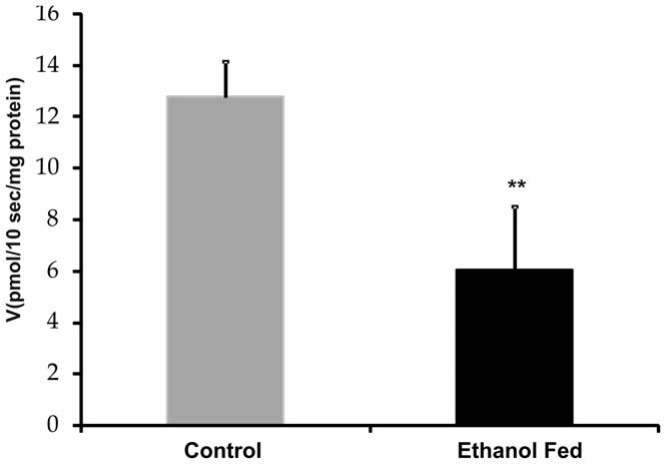
Folate uptake in pancreatic plasma membrane vesicles. Uptake of 5-[^14^C]-MethylTHF (0.5 µM) was measured in a buffer of pH 5.0 [100 mM NaCl, 80 mM mannitol, 10 mM HEPES, 10 mM 2-morpholinoethanesulfonic acid pH 5.0] for 10. Each point represents the mean±S.D. of four determinations. **p<0.01 vs. Control.

**Figure 2:**
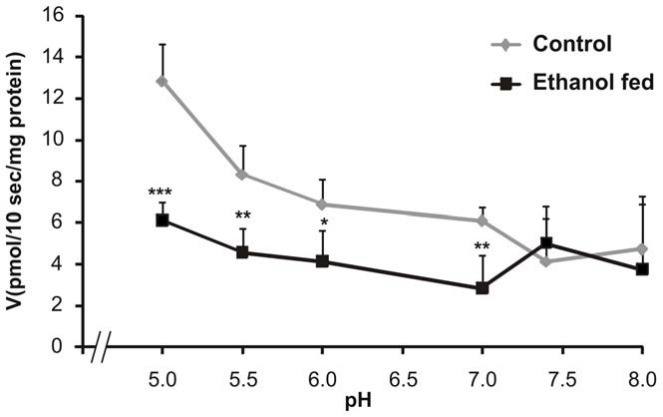
Uptake of 5-[^14^C]-MethylTHF in the rat PPMV as a function of pH optimum. Uptake was measured by varying the pH of incubation buffer [100 mM NaCl, 80 mM mannitol, 10 mM HEPES, 10 mM 2-morpholinoethanesulfonic acid from 5.0 to 8.0, keeping intravesicular pH 7.4 at 0.5 µM substrate concentration for 10 sec. Each data point is mean± SD of 4 separate uptake determinations. *p<0.05, **p<0.01, ***p<0.001 vs. control.

The folate uptake studied at 10 seconds, the measurement time selected as a time point just prior to the uptake maxima observed in both the groups (data not shown), revealed less folate uptake in ethanol fed rats as compared to control (Figure 1). In order to determine the driving force for folate transport across the pancreatic plasma membrane, pH of incubation buffer was varied from 5 to 8, keeping the intravesicular pH constant at 7.4. As shown in figure 2, with increase in hydrogen ion concentration (decrease in pH), an increase in folate uptake was observed in both the groups. This was prominently evident, when the uptake changed from neutral pH 7 to mildly acidic pH 5. Moreover, at different pH points studied, the folate uptake was 40 to 54% less (p<0.001) in ethanol fed group in comparison to control group. Notably, no such uniform decrease was observed in the alkaline pH range.

**Figure 3:**
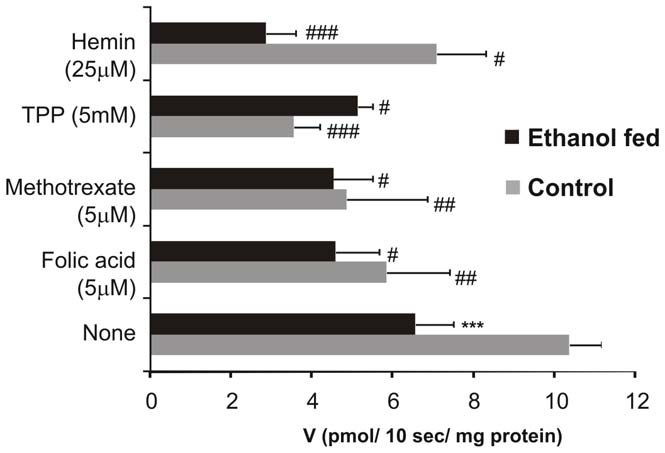
Uptake of 5-[^14^C]-methylTHF (0.5 µM) was measured with and without analog (5 µM folic acid & 5 µM methotrexate)/inhibitor {5 mM thymine pyrophosphate (TPP) & 25 µM hemin} in incubation buffer of pH 5.0. Bars are mean ±SD of 4 separate uptake determinations.

Furthermore, in order to determine the specificity of the transport system in pancreatic plasma membrane (Figure 3), the folate uptake was measured in the presence of the structural analogs methotrexate and unlabelled folic acid and inhibitors; thiamine pyrophosphate (TPP) reported to be a substrate of RFC [22] and hemin, inhibitor of folate transport via PCFT [5]. The structural analog methotrexate and folic acid decreased the transport by 52 (p<0.01) and 43% (p<0.001) in control and 30% each (p<0.05) in ethanol fed rats respectively. The inhibitor hemin decreased the transport by 31% (p<0.05) in control and 51% (p<0.01) in ethanol fed rats while the inhibitor TPP decreased the transport by 65% (p<0.001) in control and 21% (p<0.05) in ethanol fed rats respectively.

### Expression of the mRNA corresponding to *PCFT* and the *RFC* in the pancreas

**Figure 4:**
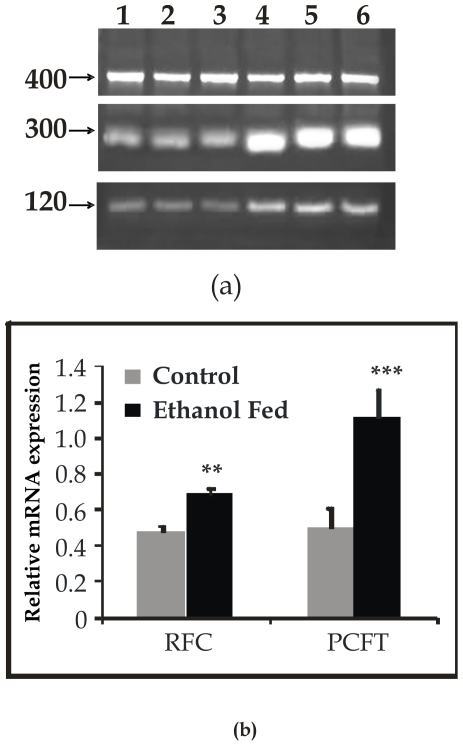
RT-PCR analysis of *RFC* (120 bp) and *PCFT* (300 bp) with *GAPDH* (400 bp) as an internal control in pancreas. (a) Resolved on 1.2% agarose gel electrophoresis and (b) densitometric analysis representing relative change in PCFT and RFC mRNA expression. Data shown are representative of 5 separate sets of experiments. Lanes 1-4: Control; 5–8: Ethanol fed. ^**^p<0.01, ^***^p<0.001 vs. Control.

In order to elucidate the mechanism of reduced folate transport in chronic alcoholism, transcriptional regulation of the *PCFT* and the *RFC* was studied. For mRNA expression, total RNA was isolated from the pancreatic tissue from both groups of rats. RT-PCR analysis was performed with the use of gene-specific primers as described in material and methods section. The relative mRNA for the PCFT was ∼2.2 fold (54%) higher (p<0.01) while that for the RFC was ∼1.4 fold (30%) higher (p<0.01) in the ethanol fed group (Figure 4). Thus, ethanol associated decrease in pancreatic folate uptake was not associated with impairment in transcriptional regulation of the PCFT and the RFC, suggesting a differential regulatory mechanism which might reduce the uptake across the PPMV.

### Expression of the PCFT and the RFC protein

**Figure 5:**
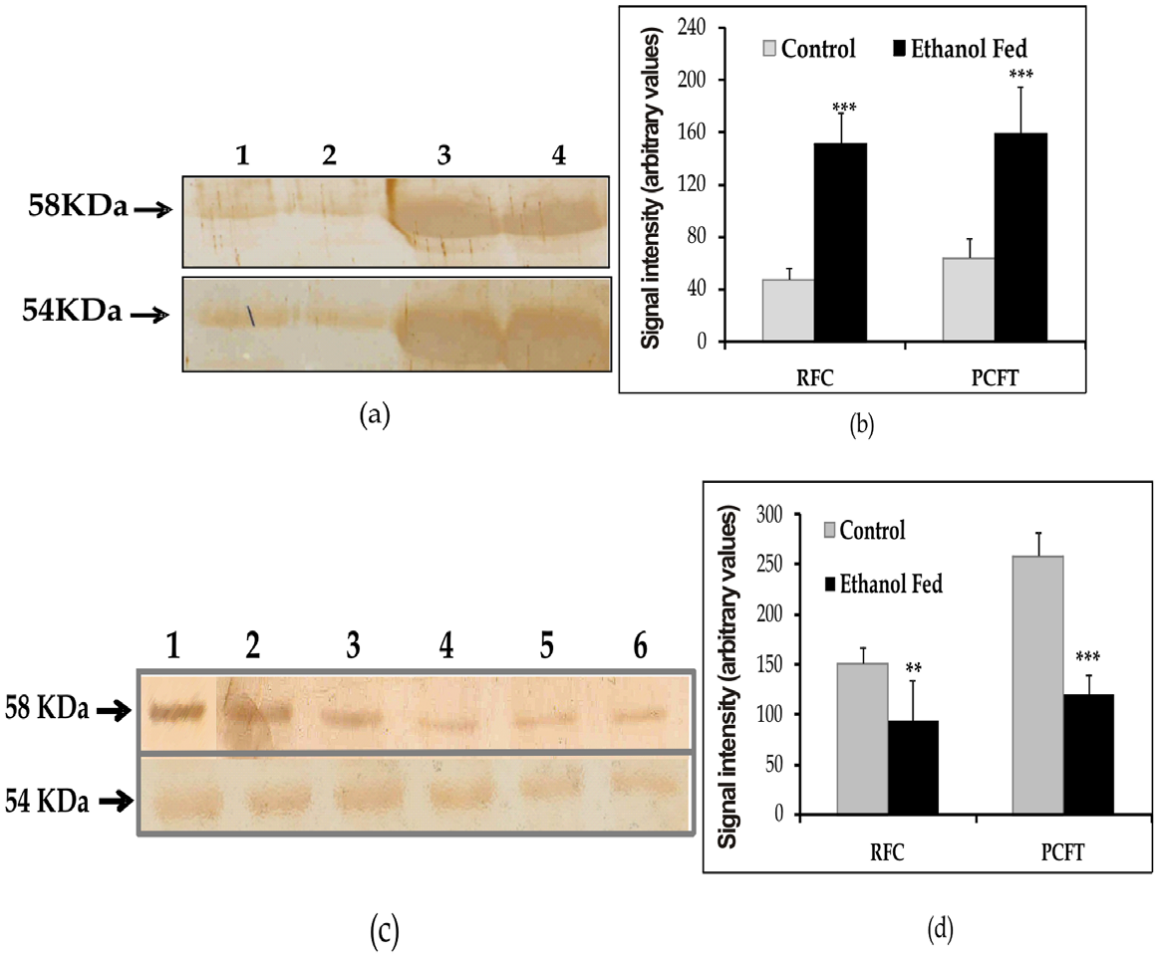
Western blots analysis of pancreatic tissue lysate (a) using anti RFC (58 kDa), anti PCFT (54 kDa) antibodies (b) Graph represents summary data of densitometric analysis, lane 1,2: Control; 3,4: ethanol fed. Western blot analysis of the PPMV (c) using anti RFC (58 kDa), anti PCFT (54 kDa) antibodies (d) Graph represents summary data of densitometric analysis. Data are expressed are means± SD of 4 separate experiments. Lane 1–3: Control; 4–6: ethanol fed. ^**^p<0.01, ^***^p<0.001 vs. Control.

The finding that the ethanol ingestion resulted in a significant increase in mRNA levels of both the PCFT and the RFC, led us to study whether this increase in mRNA levels was associated with protein levels. To investigate the effect of chronic alcohol feeding on the level of expression of the PCFT and the RFC protein, we performed western blotting on tissue homogenate and on the plasma membrane vesicles prepared from the pancreas of both the groups of rats (Figure 5). Parallel to the observed increase in mRNA expression a significant increase in the level of expression of the PCFT (p<0.05) and the RFC (p<0.01) proteins was found in the pancreatic tissues of the ethanol fed rats (Figure 5a&b). However, the level of expression of the PCFT and the RFC on the PPMV (Figure 5c&d), revealed a decrease of 1.75 fold (42%) for the PCFT and 2 fold (51%) for the RFC in the ethanol fed rats as compared to the respective controls (p<0.001, p<0.01).

### Association of the folate transporters (PCFT and RFC) with lipid rafts

To find out whether the folate transporters (PCFT and RFC) are associated with lipid microdomains of the pancreas plasma membrane of rat, partitioning of the PCFT and RFC between detergent soluble (DS) and detergent insoluble (DI) fractions of rat pancreas plasma membrane was performed, which revealed the presence of both PCFT and RFC in the DI fractions of the pancreas plasma membrane (data not shown).

**Figure 6:**
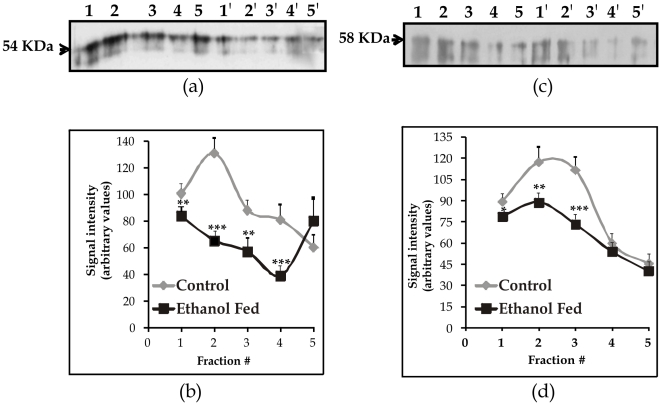
Association of folate transporters (PCFT & RFC) proteins with lipid rafts in pancreatic apical membrane. The pancreatic plasma membrane vesicles were subjected to floatation on Optiprep density gradients, and fractions were collected from top of the gradients (fractions 1–4 represent detergent-resistant membrane). Fractions were separated by electrophoresis and analyzed by Western blotting using a) anti-PCFT (54 kDa) and b) RFC (58 kDa) antibodies. Blots were scanned, and the intensity of bands was determined by densitometric analysis. Data are means±SD of 4 separate experiments. The representative blot shown for PCFT and RFC expression as, lane 1–5: Control; lane 1′–5′: ethanol fed. ^*^p<0.05^ **^p<0.01, ^***^p<0.001 vs. Control.

We next examined the distribution of the PCFT and the RFC of rat pancreas plasma membrane on Optiprep density gradient. We have validated this technique earlier by measuring the specific activity of alkaline phosphatase (well-known marker for lipid rafts) in all the fractions collected from gradient using colon apical membranes [20]. The pattern of specific activity of alkaline phosphatase in all these fractions revealed a gradient with considerable activity in the top floating fractions (1–4), indicating that these fractions contained lipid rafts. So the fractions isolated from the gradient using the pancreatic plasma membrane were subjected to western blotting for the PCFT and the RFC (Figure 6). We found the presence of the PCFT and RFC protein in the top 5 floating fractions (20– 30% Optiprep gradient) with negligible or no expression thereafter of Optiprep density gradient. Together, these data provide strong evidence that the majority of the PCFT and the RFC pool in the pancreas plasma membrane are associated with the DI lipid raft microdomains. Moreover, there was less expression of both the PCFT and the RFC in the lipid rafts in chronic alcoholism (Figure 6). The extent of decrease was 25 to 45% for PCFT (p<0.001) and 15 to 46% for RFC (p<0.01) respectively, which is in accordance with the decreased levels of these transporters in the PPMV.

### Localization of PCFT and RFC in pancreas

**Figure 7:**
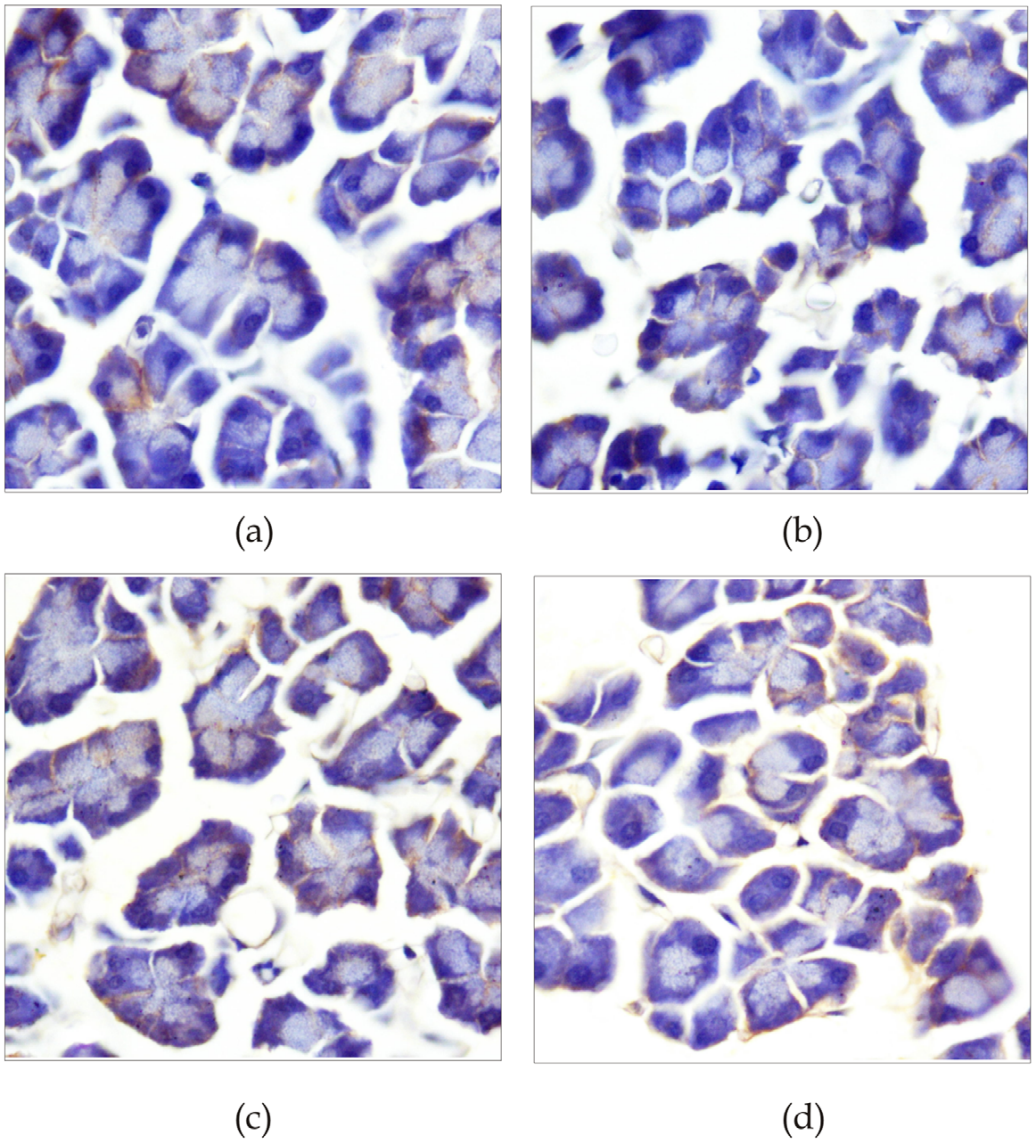
Immunohistochemical analysis of rat pancreas sections exposed to anti-PCFT and anti-RFC antibodies showing relative localization of PCFT (a&b) and RFC (c&d) protein. Figures (100x) shown are representative of each group.

Studying the localization of these transporters in pancreas by immunohistochemistry (Figure 7) revealed the localization of the PCFT (Figure 7a&b) and RFC (Figure 7c&d) at the basolateral side of pancreatic plasma membrane. In ethanol fed rats there was a marked reduction in the intensity of RFC and PCFT positive cells in pancreas.

### Serum, RBCs, and tissue folate levels

As this study dealt with hindered folate absorption during alcoholism, the determination of folate levels was of prime importance. The results showed that a significant decrease (p < 0.001) in serum, RBCs (p<0.001) and pancreatic tissue (p<0.01) folate levels in chronic alcoholism (Table 1), confirming the association of alcoholism with folate deficiency. Due to the limitations of the method employed to measure released folate from the tissue, the tissue folate levels presented here are a comparison of relative folate extracted from the control and ethanol fed rats and these levels may not necessarily reflect the true pancreatic folate.

**Table 1:** Folate levels in serum, RBCs and pancreatic tissue of control and ethanol fed rats

	Control	Ethanol fed
Serum folate ( µg/L)	49.64± 3.2	33.71±5.6^***^
RBC folate ( µg/L)	950±24.9	624±10.5^***^
Pancreas (nmol/g wet tissue)	9.90±0.95	7.92±0.32^**^

Values are mean±SD (n = 6).**p<0.01, ***p<0.001 vs control

### Methylation of the *PCFT* and the *RFC* gene

**Figure 8:**
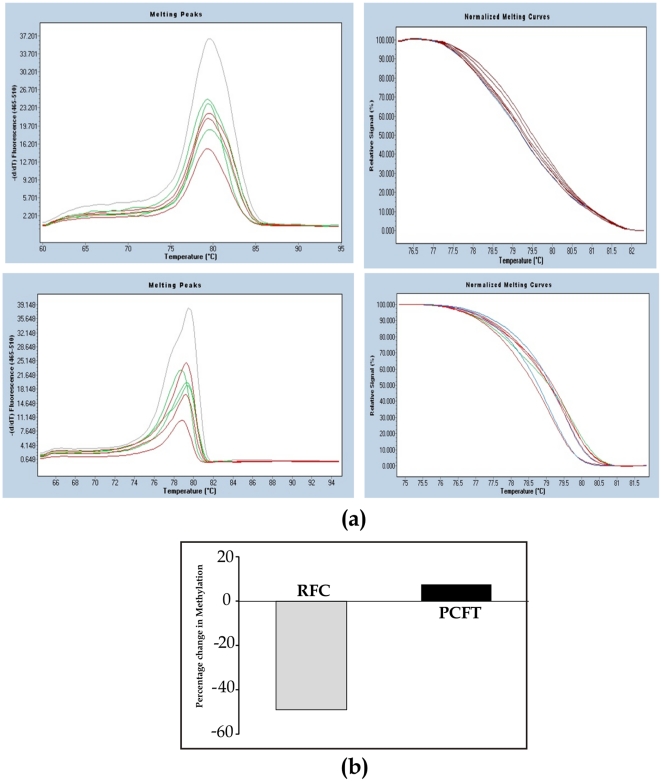
HRM analysis of PCFT and RFC gene (a) upper panel PCFT lower panel RFC, upper/lower panel left side melting profile and upper panel/lower panel right normalized melting curves. Curves in green; Control, red; Ethanol fed and gray; 100% methylated. (b) Percentage change in methylation of genes in ethanol fed rats compared to control. ^***^p<0.001 vs. Control.

It was of interest to evaluate that how alcohol associated reduced folate levels in the pancreas would affect the promoter region methylation of the folate transporters. Hence we determined the CpG island methylation of PCFT and *RFC* gene. As shown in Fig 8 there was a significant decrease (48%) in methylation of promoter region of *RFC* gene in ethanol fed rats as compared to control (Figure 8b), however no significant change in methylation of PCFT promoter region was observed.

## Discussion

Ethanol causes a wide spectrum of health problems, as a consequence of altering several metabolic pathways in every organ of the body [23]. Studies have found that the pancreas is sensitive to the effect of ethanol and its metabolites [24]. Moreover, several observations suggest that one-carbon compounds may play a special role in the function of the exocrine pancreas involving synthesis and secretion of a number of enzymes necessary for the digestion of macronutrients. Folate being central to methyl group metabolism is therefore essential for the normal function of the exocrine pancreas [8], [10]–[12], [25]. Despite the importance of folate for the exocrine pancreas, little is known about the regulation of folate transport across the PPM and interference in uptake by ethanol consumption. Despite its direct effects, the ethanol might impair the functions of the pancreas by associated folate deficiency and hence impaired one carbon metabolism. Keeping in view, the present study was designed to characterize the folate uptake by the PPMV, and the role of regulatory folate transport mechanisms in an *in vivo* model of chronic alcoholism. Earlier studies in our laboratory have demonstrated that a significant concentration of blood alcohol was maintained after the last dose of ethanol of (1 g/kg) body weight per day at the end of a 3 month course [4]. Such a dose was chosen according to earlier studies [26], which suggested that the ethanol concentration of jejunal tissue should not exceed 6% in animal experiments in order to be relevant to the humans. Moreover, such relevance is also based on the fact that when a 0.8 g/kg of acute ethanol was administrated orally in the experimental animals; levels of ethanol greater than 0.4% are maintained for up to 60 min in blood [27]. In such a dose, the peak level in duodenum and proximal jejunum is between 1 and 5% respectively. Further, it was observed that 45–60 g ethanol in 20% solution ingested by humans, exhibited between 6.5 and 9.4% in the duodenum and between 5.7 and 6.4% levels of ethanol concentration in the jejunum. Thus, in the present study, 1 g/kg body weight/day ethanol (20%) is expected and developed blood alcohol concentrations at nontoxic values.

The activities of folate transporters on the pancreatic plasma membranes play an important role in uptake of folate across pancreatic plasma membrane, which is second (first being liver) largest storing organ of folate [7]. The pancreatic plasma membrane (PPM) mainly contains the reduced folate carrier (RFC) and proton coupled folate transporter (PCFT), which are responsible for the folate transport therein [5], [21]. Like the liver, the pancreas also possesses the enzymes responsible for ethanol metabolism. Therefore, pancreas is susceptible to the toxic effects of ethanol and its metabolites [28]–[30].

In the present study ethanol ingestion in rats led to a significant decrease in serum, RBCs, and pancreatic folate levels, suggesting strong association of chronic alcoholism with folate deficiency. The acidic pH was found to be a driving component of the folate uptake across the PPM as the maximal uptake was obtained at the pH of 5.0, showing similar characteristics as of folate transport across intestinal, renal and colon apical membranes [5], [17], [19], [20], [31]. The existence of acidic pH at the PPM surface is due to the presence of H^+^-ATP_ase_ pump which generates H+ gradient [32]. The observed reduction in folate uptake in ethanol fed group was not uniformly apparent in alkaline pH range which further suggested that the importance of acidic pH in the operation of folate transport process.

Different structural analogs such as folic acid and methotrexate decreased the folate uptake in PPMV hence validating the specificity of the transport systems. The observed decrease was less in ethanol fed rats than control, suggesting that affinity of folate transporters to other substrates might have also got perturbed. The presence of hemin (inhibitor of PCFT) and TPP (inhibitor of RFC) significantly reduced the folate transport across the PPM, suggesting that both these transporters are involved in folate uptake in pancreas in comparison to that observed in the CAM where PCFT is mainly responsible for the uptake [20].

To know the molecular mechanisms of reduced folate uptake in ethanol fed rats, the expression of the transporter molecules was important to study. The mRNA expression studies revealed an increase in expression of folate transporters in pancreatic tissue in chronic alcoholism, which was associated with increased expression of transporter proteins in pancreas. However, western blot analysis of PPM revealed the decreased expression of transporters in PPM which explained the observed decrease in folate transport activity in ethanol fed rats. Our results regarding decreased pancreatic folate uptake in ethanol fed rats are in agreement to that of Said et al. [21] who have also shown a reduced folic acid uptake by pancreatic acinar cells isolated from rats fed alcohol chronically. However, increased expression of RFC and PCFT in ethanol fed rats observed in our study is in contradiction to their study in which they have found reduced expression of folate transporters in pancreatic acinar cells of rats during chronic alcoholism. Such differences might be due to use of pure pancreatic acinar cells in their study [21] and pancreatic tissue extract in our study. This suggests that there might be decrease in expression of folate transporters in acinar cells and increase expression in other cells. Moreover such differences might be also due to differences in the dose and duration of ethanol ingestion and the strain of the rats used in the two studies. The upregulation of transporters as observed by us, in the pancreas of rats after chronic ethanol consumption can be explained by the earlier observations on increased ATF3 expression in the tissue in chronic alcoholism. Besides ATF3 many genes were found to be upregulated which included heat shock protein 70, heat shock protein 27 and mesotrypsinogen. The mechanism of increased gene expression can be explained by upregulation of ATF3 expression which regulates ER stress regulated kinases. Upon ER stress or protein load, these kinases inactivate eukaryotic initiation factor (eIF2α) by phosphorylation [28], thereby inhibiting protein synthesis. ATF3 activates phosphatases which inactivate ER stress kinases thus releasing the protein translational block and increasing the protein synthesis for maintaining cellular homeostasis and for the cells to respond to further stress [33].

The observed poor folate absorption across the PPM during alcoholism in the present study cannot be ascribed to reduced protein synthesis so we hypothesized that some posttranslational modifications or deranged trafficking event might have resulted in reduced levels of folate transporters in PPM. To look into this aspect, we determined the association of these transporter proteins with membrane LRs. Lipid rafts (LRs) are the specialized microdomains of the plasma membrane that are essential for the normal functioning of various membrane transporters [34]–[36]. Proteins might enter LR at the Golgi level and their shuttling between the Golgi and cell membranes allows cell to exert regulatory control over the surface expression of their proteins. There is no report regarding the association of the PCFT and the RFC with lipid rafts in the PPM. We first examined the distribution of PPM utilizing Optiprep floatation; we found that folate transporter proteins; PCFT and RFC were present in the floating fractions corresponding to lipid raft microdomains of pancreatic plasma membrane vesicles. Our findings, regarding the presence of PCFT and RFC in LR of the PPM of rats in agreement to our earlier studies in the CAM [20], suggest the alteration of the lipid composition of pancreatic plasma membranes might result in disruption of LR in chronic alcoholism. The reduced levels of the PCFT and RFC in the PPM as compared to that in the whole cell lysates in ethanol fed rats might be due to reduced association of these proteins with LR at the PPM or alternatively reflect the role of post-translational/or trafficking event that regulates the number of transporter molecules in the LR of the PPM during alcoholism. However, further studies need to be addressed to know the exact mechanisms. In accordance with the immunoblot analysis, immunohistochemical staining of pancreatic tissue revealed the PCFT and RFC localization to the basolateral side of pancreatic plasma membrane.

Low folic acid status is often associated with impaired DNA methylation [37], affecting gene expression in complex ways [38]–[40]. We sought to determine how the DNA methylation of the folate transporter genes PCFT and RFC is affected under conditions of reduced pancreatic folate status observed in ethanol fed rats. We observed hypomethylation in CpG island of RFC but not of PCFT gene in ethanol fed group. These observations suggest that the effect of decreased folate on the DNA methylation in the pancreas is gene specific [38], [41]. However the role of the direct effects of ethanol on DNA methylation under these conditions cannot be ruled out [1], [42]–[45]. Moreover, these results on the differential effect of DNA methylation of RFC and PCFT suggest the distinct mechanisms of regulation of the two transporters in the pancreas under the conditions of chronic alcoholism.

In conclusion, the results show that chronic ethanol ingestion leads to decreased pancreatic folate uptake. The reduced association of the PCFT and RFC with LR at the PPM was associated with the decreased folate uptake across the PPM thereby leading to reduced pancreatic tissue folate levels. The consequences of which was aberrant DNA methylation of RFC. However, further studies are needed to delineate the exact molecular events which could explain the role of the post-translational and/or the trafficking events that regulate the number of transporter molecules in the PPM during chronic alcoholism.

## Materials and Methods

### Chemicals

Radiolabelled (6S) 5-[^14^C]-methyltetrahydrofolate, potassium salt with specific activity 24.0 Ci/mmol were purchased from Amersham Pharmacia Biotech (Hong Kong). Color burst^TM^ electrophoresis marker (M.W.8,000–220,000) was purchased from Sigma Chemical Co., St. Louis, MO, USA. Total RNA Extraction Kit was purchased from Taurus Scientific, Cincinnati, USA. Moloney Murine Leukemia Virus reverse transcriptase (RevertAid^TM^ M-MuLV RT) kit was purchased from the MBI Fermentas, Life Sciences, USA. RNA later (RNA stabilization solution) were obtained from Ambion, Inc. Austin, USA. Primary antibodies rabbit anti rat RFC (RFC) and anti rat PCFT (PCFT) polyclonal antibodies were raised in rabbits in our laboratory [46]. HRP labeled goat anti rabbit-IgG secondary antibodies was purchased from G Biosciences, St Louis, MO, USA. Enhanced chemiluminescence detection kit was purchased from Biological industries ltd. Kibbutz beit Haemek, Israel. Metal enhanced DAB substrate kit was purchased from Thermo Fisher Scientific Inc, Rockford, USA. LightCycler® 480 High Resolution Melting Master Mix was purchased from Roche. Cryoprotected *L. casei* bacterial strain (MTCC 1423) was purchased from IMTECH, Chandigarh, India.

### Animals

Young adult male albino rats (Wistar strain) weighing 100–150 g were obtained from Institute's Central Animal House. The rats were housed in clean wired mesh cages with controlled temperature (23±1°C) and humidity (45–55%) having a 12 hr dark light cycle throughout the study. The rats were randomized into two groups of 8 animals each, such that the mean body weights and the range of body weights for each group of animals were similar. The rats in group I were given 1 g ethanol (20% solution)/kg body weight/day and those in group II received isocaloric amount of sucrose (36% solution) orally by Ryle's tube daily for 3 months. The rats were fed a commercially available pellet diet (Ashirwad Industries, India) containing 2 mg folic acid per kg diet and water *ad libitum*. The body weights of rats were recorded twice a week. Overnight fasted rats were sacrificed under anesthesia using sodium pentothal.

The protocol of the study was approved by “Institute Animal Ethics Committee” (40/IAEC/149) Postgraduate Institute of Medical Education and Research, Chandigarh, India and “Institutional Biosafety Committee” (IBC-2008/173) Postgraduate Institute of Medical Education and Research, Chandigarh, India.

### Preparation of pancreatic plasma membrane vesicles (PPMV)

Pancreatic plasma membranes vesicles (PPMV) were prepared from rat pancreas by using the method by Robert C. De Lisle [47], with some modifications. Rat pancreatic tissue was minced with scissors and suspended in homogenization buffer [1 mM NaHCO_3_, pH7.4, 0.5 mM MgCl_2_, 0.1 mM phenylmethylsulfonyl fluoride]. The suspension was homogenized with a glass-teflon homogenizer at 1300 rpm for 1 minute. The homogenate was centrifuged to pellet unbroken cells by accelerating the centrifuge to 1300xg and then stopping immediately. The pellet was rehomogenized and pelleted as above and the two supernatants were combined. The combined supernatants were considered as the homogenate which was then diluted 20-fold in the same buffer without MgCl_2_ containing 0.7 mM EDTA, and incubated on ice for 10 min. The homogenate was filtered through four layers of gauze and was centrifuged at 1300xg for 15 min to pellet large membrane sheets. The upper surface of the pellet (the “fluffy” layer) was rinsed off with homogenization buffer and the remaining pellet was considered as the crude plasma membrane fraction. The crude plasma membranes were resuspended in 46% sucrose (w/v) in a dounce homogenizer with a tight pestle, placed into centrifuge tubes, overlaid with 10% sucrose, and centrifuged for 2 h at 38,000 rpm (sorvall-M150 SE). For the preparation of PPMV, the purified plasma membranes were removed from the interface, centrifuged at 38,000 rpm for 30 minutes in loading buffer containing [280 mM mannitol, 20 mM HEPES-Tris, pH 7.4]. The resulting pellet resuspended in same buffer at ∼5 mg protein per mL.

Purity of the membrane preparations was checked by measuring the specific activities of Na^+^, K^+^-ATPase (basolateral membrane marker) in the PPMV and in original homogenate by the method of Quigley and Gotterer [48] and γ-glutamyltranspeptidase (apical membrane marker) by the method of Orlowski and Meister [49]. The vesicle preparations from both the groups showed enrichment of 15–19-fold (90–95%) with respect to Na^+^, K^+^-ATPase activity and 2–3-fold (5–6%) with respect to γ-glutamyltranspeptidase. These preparations were thus primarily of basolateral origin.

### Transport of 5-[^14^C]-methyltetrahydrofolate

Uptake studies were performed at 37°C using the incubation buffer of 100 mM NaCl, 80 mM mannitol, 10 mM HEPES, 10 mM 2-morpholinoethanesulfonic acid, pH 5.0 and 0.5 µM of 5-[^14^C]-methyltetrahydrofolate, unless otherwise mentioned. The initial rate of reaction was measured by stopping the transport reaction at 10 seconds, and that this rate was not corrected for any initial rapid folate binding. Ten µL of isolated pancreatic plasma membrane vesicles (50 µg protein) from the control and the ethanol fed rats for different specific assays were added to incubation buffer containing 5-[^14^C]-methyltetrahydrofolate of known concentration. Reaction was stopped by adding ice-cold stop solution containing 280 mM mannitol, 20 mM HEPES-Tris, pH 7.4 followed by rapid vacuum filtration. Non-specific binding to the filters was determined by residual filter counts after filtration of the incubation buffer and labeled substrate without vesicles, as described earlier [1], [4]. The radioactivity retained by the filters was determined by liquid scintillation counting (Beckman Coulter LS 6500).

### Reverse transcriptase (RT)-PCR analysis

Total RNA was isolated from the pancreas by using total RNA extraction kit and cDNA synthesis was carried out from the purified and intact total RNA according to manufacturer's instructions. Expression of *RFC*, *PCFT,* and *GAPDH* was evaluated using sequence specific primers corresponding to the sequence in the open reading frame. PCR mixture (20 µL) was prepared in 1x PCR buffer consisting of 0.6 U of Taq polymerase, 2 µM of each primer for *GAPDH*, *PCFT* and *RFC* along with 200 µM of each dNTP. In optimized PCR, the initial denaturation step was carried out for 2 min @ 95°C. The denaturation, annealing, and elongation steps were carried out respectively for 1 min@94°C, 45 sec @ 64°C (*PCFT*) or 56°C (*GAPDH)* and 1 min @72°C for 35 cycles. In case of *RFC* denaturation, annealing and elongation steps were carried out respectively for 30 sec@94°C, 30 sec@52.1°C, 30 sec@72°C for 35 cycles. The final extension step was carried out for 10 min@72°C. The primers were designed using Primer3 Input (version 0.4.0). The sequences of the primers used were as follows: 5′-CATGCTAAGCGAACTGGTGA-3′(sense) and 5′-TTTCCACAGGACATGGACA-3′ (antisense) for RFC, 5′-AAGCCAGTTATGGGCAACAC-3′ (sense) and 5′-GGATAGGCTGTGGTCAAGGA-3′ (antisense) for *PCFT*, and 5′-CCTTCATTGACCTCAACTACAT-3′ (sense) and 5′-CCAAAGTTGTCATGGATGACC-3′ (antisense) for *GAPDH*. The expected PCR products of size 120, 300 and 400 bp were obtained for *RFC*, *PCFT* and *GAPDH* respectively when electrophoresed on 1.2% agarose gel. The densitometric analyses of products were determined by using ‘Scion image’ software.

### Western blot analysis

For protein expression studies, PPMV (100 µg) were resolved on 10% SDS-PAGE and transferred to PVDF membrane for 20 minutes at 15 V. Western blotting was performed using the procedure described by Towbin [50]; using polyclonal primary antibodies as rabbit anti-rat RFC (1:800 dilutions) raised against specific region of rat RFC synthetic peptide corresponding to amino acids 494–512 [51]. The polyclonal antibodies against PCFT (1:1000 dilutions) were raised against specific region of rat PCFT synthetic peptide corresponding to amino acids 442–459. Secondary antibodies used were goat anti-rabbit IgG-HRP-labeled (1:20,000 dilutions). The bands were visualized by either metal enhanced DAB substrate kit or enhanced chemiluminescence detection kit according to the manufacturer's instructions. The quantification of blots was carried out by using ‘Scion image’.

### Isolation of detergent-soluble and detergent-insoluble fractions from rat pancreas plasma membrane vesicles

Detergent-soluble (DS) and insoluble (DI) fractions of PPMV were prepared essentially as described by [52]. Briefly, 3 mg of PPMVs were centrifuged for 30 min at 100,000x*g* at 4°C and suspended in MES buffer containing 50 mM MES (pH 6.5), 60 mM NaCl, 3 mM EGTA, 5 mM MgCl_2_, 1% Triton X-100, and 1xcomplete protease inhibitor cocktail. Membrane vesicles were then incubated with MES buffer on a rotary shaker for 30 min at 4°C. At the end of the incubation, PPMVs were centrifuged at 100,000x*g* at 4°C for 30 min, and supernatant was designated as DS fraction. The pellet was resuspended in buffer containing 15 mM HEPES (pH 7.4), 150 mM NaCl, 10 mM EDTA, 1 mM DTT, 1% Triton X-100, 0.1% SDS, and 1x complete protease inhibitor cocktail and was designated as DI fraction. Both DS and DI fractions were analyzed by western blotting.

### Floatation on a discontinuous Optiprep density gradient

Lipid rafts were isolated by floatation on Optiprep density gradient [53]. Although this protocol is widely used for isolating lipid rafts, yet the protocol has limitations as localization to detergent resistant membrane fractions is not an accurate criterion for assigning raft residency. Briefly, pancreatic plasma membrane vesicles were centrifuged at 100,000x*g* for 30 min at 4°C and then resuspended and incubated for 30 min at 4°C in TNE buffer containing 25 mM Tris (pH 7.4), 150 mM NaCl, 5 mM EDTA, and 1% Triton X-100 supplemented with 1x complete protease inhibitor cocktail. The membranes were then adjusted to 40% final concentration of Optiprep (Sigma Aldrich, USA) and layered at the bottom of density gradient with steps of final concentrations of 35%, 30%, 25%, and 20% of Optiprep in TNE buffer. TNE buffer was laid on the top of the gradient, which was then centrifuged at 215,000x*g* for 4 h at 4°C. Fractions were collected from the top of the gradient and then analyzed by Western blotting. Proteins in the top four fractions are considered to be raft-associated [54]. We are referring these fractions as lipid rafts in the manuscript however, these might not represent the actual LRs due to limitations of the protocol. Protein concentrations in each fraction were assessed by using a Bradford kit.

### Immunohistochemical analysis

Freshly removed pancreas was cut followed by fixing in a sufficient amount of 10% formalin [55]. Sectioning on poly-L-lysine coated slides & fixated at 37°C overnight. Endogenous peroxidase was quenched by pretreatment with 1% H_2_O_2_ in methanol for 20 min followed by washings in PBS. Slides were put in primary diluted antibody (rabbit polyclonal anti- rat RFC and PCFT [1:200] for 2 hrs at 37°C followed by secondary antibodies as [goat anti-rabbit IgG-HRP (1:200) and]. Presence of antibody at specific site(s) was revealed using freshly prepared 3,3′-diaminobenzene & H_2_O_2_ at room temperature for 3–5 min & counter staining with hematoxylin.

### Estimation of folate by microbiological assay

The folate estimations were determined by microtitre plate assay using *Lactobacillus casei* [56] as described earlier [57]. For intracellular folate concentrations in pancreas, a 10% homogenate of pancreas was made in phosphate buffer of pH 6.3 containing 5 mg/mL ascorbate. The homogenate was incubated at 110°C for 10 minutes followed by centrifugation at 300 rpm for 10 minutes. The supernatant (0.1 mL) was then treated with 0.02 mL of rat plasma conjugase in 0.375 mL of phosphate buffer of pH 4.5 [58]. For standardizing the time point of treatment with conjugase enzyme, the folate hydrolase treatment was carried out by incubating the tissue extract with enzyme (folate hydrolase) in presence of ascorbic acid for 1 to 5 hours at 37°C and the reaction was stopped by freezing the mixture. The released folate levels increased till 4 hours incubation with no change in the levels thereafter. Hence, 4 hour time was chosen for further experiments. Folate levels in the samples were corrected for the levels measured in 0.02 mL of plasma folate hydrolase. The free folate was determined by a standard microbiological microtitre plate assay using *Lactobacillus casei.* All the steps were carried out in aseptic conditions.

### Methylation of DNA using high-resolution melting (HRM)

#### DNA isolation

DNA isolation was carried out from the pancreatic tissue by using the DNA isolation kit (Real Genomics^TM^) according to manufacturer's instructions.

#### High-resolution melting (HRM) analysis

PCR amplification and HRM were performed on the LightCycler® 480 Instrument (Roche) as adapted from the published protocol by Wojdacz and Dobrovic [59]. Primer sequences included no more than 1 to 2 CpG sites which were placed at or adjacent to the 5′ end [60]. Oligonucleotide sequences were as follows: 5′-TAGGTCGTGGTGTTTATTTTTG-3′ (sense) 5′-AAAAAAACCGTTAAAATCTCCC-3′ (antisense) for *PCFT* and5′-GTTTGCGAAGAGTTTAGGTAGG-3′ (sense) 5′-TCGCAACTATACCATAAAACCA-3′ (antisense) for *RFC*. A fully (100%) methylated DNA control was prepared by methylating the rat genomic DNA in vitro with the CpG methylase enzyme SssI (New England Biolabs). The control DNA as well as DNA isolated from pancreatic tissues were treated with bisulphite using the EZ DNA Methylation Gold^TM^ kit (Zymo research, Orange, CA, USA) according to the manufacturer's instructions. PCR was carried out in a final volume of 20 µL containing: 1x LightCycler® 480 High Resolution Melting Master mix, 150 nmol of each primer for *PCFT* and RFC, 80 ng bisulfite treated DNA template, with 3 mM final MgCl_2_. Each reaction was performed in duplicate. The cycling conditions were as follows: 10 min at 95°C, 50 cycles of 20 s at 95°C, 30 s at primer specific annealing temperature, and 30 s at 72°C. The PCR products were subjected to HRM scans using the following conditions: 95°C for 1 min, 40°C for 1 min, 65°C for 1 s, and continuous acquisition to 95°C at 22 acquisitions per 0.04°C. The normalized HRM profiles allow estimation of methylation levels of unknown samples run along with the 100% methylated standard.

### Statistical analysis

Each uptake assay was performed thrice with 4 independent preparations from each group. Statistical analysis was performed with Graphpad Prism software (Avenida de la Playa La Jolla, USA). The data was computed as mean±SD. Group means were compared by using Student's *t*-test and ANOVA. The acceptable level of significance was 5% for each analysis.
